# Formation and Identification of a 5-(Hydroxymethyl)-2-Furfural-Zingerone Condensate and Its Cytotoxicity in Caco-2 Cells

**DOI:** 10.3389/fnut.2022.893991

**Published:** 2022-04-28

**Authors:** Yujing Ke, Zhao Yin, Nenghua Chen, Peifang Chen, Jie Liu, Shiyi Ou, Guoqiang Li

**Affiliations:** ^1^Department of Food Science, Foshan University, Foshan, China; ^2^Department of Hematology, Guangdong Second Provincial General Hospital, Guangzhou, China; ^3^Department of Food Science and Engineering, Jinan University, Guangzhou, China; ^4^School of Medicine, Shenzhen University, Shenzhen, China; ^5^South China National Center for Food Safety Research and Development, Foshan, China

**Keywords:** 5-(hydroxymethyl)-2-furfural, zingerone, adduct, aldol condensation, cytotoxicity

## Abstract

5-(Hydroxymethyl)-2-furfural (HMF), an active furfural, widely exists in various food products and has potential safety risks. It can be eliminated by occurring aldol condensation with α-unsubstituted ketones in the presence of catalysts. However, the elimination process between HMF and ketones from food is rarely studied. In this study, the adduct formation between HMF and zingerone (ZGR) catalyzed by proline was investigated. It revealed that the adduct formation led to 99.75% of HMF being trapped under the optimized reaction condition. Moreover, the *in vitro* digestion stability of HMF-ZGR adduct (HMZ) and its cytotoxicity against Caco-2 cells were evaluated. The results indicated that more than 75% of HMZ was remained after a three-stage simulated digestion. Following 24 and 48 h of incubation, HMZ exhibited cytotoxicity against Caco-2 cells with IC_50_ values of 41.47 ± 5.33 and 25.39 ± 3.12 mM, respectively, versus 35.39 ± 4.03 and 19.17 ± 2.10 mM by HMF.

## Graphical Abstract

***In vitro*** **digestion of the adduct (HMZ) formed between zingerone and HMF, cytotoxicity against Caco-2 cells of HMZ, and absorption of HMZ by Caco-2 cells**. 
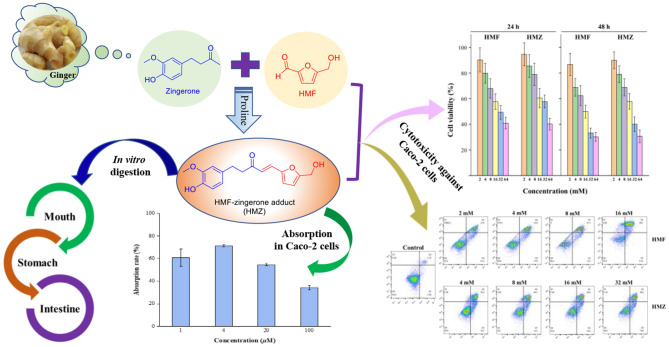

## Introduction

5-(Hydroxymethyl)-2-furfural (HMF), a common heat-induced endogenous compound, is formed as an intermediary product of Maillard reaction or carbohydrate dehydration in a low pH environment (1). It exists in a wide range of food products, such as honey (e.g., mean HMF concentration in Malaysian honey at 4–5°C to be 35.98 mg/kg after 2 months storage while that at 25–30°C to be 118.47–1139.95 mg/kg after 1 year storage) (2), coffee (e.g., 213.02–238.99 and 336.03–362.05 mg/kg HMF in traditionally and instant Turkish coffee before brewing, respectively) (3), cereal or cereal products (e.g., breakfast cereals, 6.91–240.51 mg/kg) (4), dried fruits (e.g., dried plum, 1,600–2,200 mg/kg) (5), and dairy products (e.g., infant milk powder, mean 2.3 mg/kg) (6). However, it is found that HMF could exert negative effects on human beings. For example, high HMF concentration can irritate eyes, upper respiratory tract, skin, and mucous membranes (7). On the other hand, HMF can also metabolize into toxic 5-sulfo-oxymethylfurfural (SMF) *in vivo*, of which mutagenicity has been recognized by the European Food Safety Authority (EFSA) (8, 9). Additionally, Bakhiya et al. confirmed that SMF could induce nephrotoxicity after the uptake by cells (10). Besides, SMF also caused hepatic lesions in a study carried out on FVB/N mice, in which the mice were administrated with SMF at dosages of 31.25, 62.5, 125, and 250 mg SMF/kg (11). Concerning the potentially harmful impact, an intake threshold of 0.54 mg/day for furan derivatives used as flavoring agents is established in Europe (12, 13). In this case, exploring effective methods to control the content of dietary HMF is of necessity.

Currently, a number of researches on adducting HMF have been conducted to find effective methods to control or reduce the HMF content in foods. For instance, Wang et al. found that Lysine could adduct HMF to form a Schiff base (HML) and 74.70% of HMF was eliminated under the optimized condition (14), and Hamzalioglu et al. reported that the reaction of HMF with amino as well as sulfhydryl groups to form Michael adducts was another potential strategy for HMF elimination (8). Additionally, it is also found that polyphenols [e.g., (+)-catechin, (–)-epicatechin, and malvidin 3-*O*-glucoside] can behave as possible HMF scavengers by forming condensates with HMF (15). However, the adducts formation between HMF and ketones from food has not been reported in any literature.

Ginger, also known as ginger root, belongs to the Zingiberaceae family, and is commonly used as a flavoring agent in Asia because of its aromatic odor and pungent taste (16). Zingerone (ZGR) is one of the key active ketone compounds in ginger that possesses various biological activities, including anti-oxidant (17), anti-inflammatory (18), anti-cancer (19), anti-hyperlipidemic (20), anti-bacterial (21), and liver protection (22) effects. Proline, an amino acid commonly distributed in plants, is also widely used as an effective catalyst for the aldol reaction between ketones and aldehydes (23–25). Hence, in the present study, the adduct formation between HMF and ZGR was investigated, with proline as a catalyst. Herein, we report the investigation of different reaction conditions on the elimination of HMF and the formation of HMF-ZGR adduct (HMZ), the structural characterization of HMZ, and the evaluation of the stability of HMZ in simulated *in vitro* digestion as well as the cytotoxicity of HMZ against Caco-2 cell lines.

## Materials and Methods

### Materials

HMF (99%), ZGR (98%), proline (99%), and phosphate-buffered saline (PBS, 0.1 M, pH 7.4) were purchased from Sigma-Aldrich (Shanghai, China). High-performance liquid chromatography (HPLC)-grade methanol was acquired from Merck (Darmstadt, Germany). All other reagents were of analytical grade and sourced from Guangdong Guanghua Sci-Tech Co., Ltd. (Shantou, China). Simulated salivary fluid (SSF), simulated gastric fluid (SGF), and simulated intestinal fluid (SIF) were obtained from Phygene Biotechnology Co., Ltd. (Fuzhou, China). Minimum essential medium alpha (α-MEM) was purchased from Thermo Fisher Scientific, Inc. (Waltham, MA). Fetal bovine serum (FBS), non-essential amino acid (NEAA), penicillin, streptomycin, 0.25% trypsin-EDTA, Hank's balanced salt solution (HBSS), cell counting kit-8 (CCK-8) and Annexin V-fluorescein isothiocyanate (FITC) apoptosis detection kit were purchased from Lubio Science (Bern, Switzerland). Filters were from Jinteng Experimental Equipment Co., Ltd. (Tianjin, China). Sample solutions (HMF and HMZ) were dissolved with DMSO, diluted with PBS to different concentrations, and filtered through a 0.22 μm filter for cell experiments.

### Preparation of HMZ

The proline-catalyzed aldol reaction between acetone and several aldehydes was reported previously (26), while that between HMF and ZGR has not been described yet. Here, the preparation of the adduct between HMF and ZGR was performed as follows: ZGR (1.1 mmol), proline (0.1 mmol), HMF (1.0 mmol), and PBS (5 mL) was mixed and reacted in a three-necked flask at 37°C for 2 h. At the end of the reaction, the mixture was extracted with ethyl acetate and purified by silica column chromatography using petroleum ether/ethyl acetate (60/1, v/v) as eluent to obtain the product, HMZ.

### Determination and Characterization of HMZ

The reaction mixture and purified product were subjected to HPLC analysis (Shimadzu, Japan; with diode array detector). The sample (0.5 mg/mL, 10 μL) was dissolved with HPLC-grade methanol, filtered through a 0.45 μm filter, and analyzed by a Zorbax SB-C18 column (4.6 mm × 150 mm, 5 μm, Agilent, USA) with a gradient elution composed of eluents A (0.01% acetic acid in water) and B (100% methanol) at a flow rate of 1.0 mL/min. The mobile phase started with 90% A and 10% B, then linearly increased to 100% B from 0.01 min to 30 min, and was maintained at 100% B for 10 min. The wavelength for UV detection was set in the range of 200–400 nm.

Nuclear magnetic resonance (NMR) spectroscopy was an efficient and accurate technique for characterization, which was also applied in metabolomic analysis (27). In the present study, the chemical structure of HMZ was elucidated by collecting its 1D and 2D NMR data. HMZ (20 mg) was dissolved in 0.5 mL of CDCl_3_ at 25°C to record the ^1^H NMR, ^13^C NMR, distortionless enhancement by polarization transfer 135 (DEPT-135), 2D ^1^H correlation spectroscopy (^1^H-^1^H COSY), ^1^H-detected heteronuclear single-quantum coherence (HSQC), and ^1^H-detected heteronuclear multiple-bond correlation (HMBC) spectra on a Bruker 400 MHz NMR apparatus (Bruker Corp, Fallanden, Switzerland). Chemical shifts were expressed in parts per million (ppm).

The mass spectrometric (MS) data of HMZ was acquired using the LCMS-8045 triple quadrupole mass spectrometer (Shimadzu Corporation, Tokyo, Japan) equipped with an electrospray ionization (ESI) source in negative ionization mode. The scan range was from *m*/*z* 50 to *m*/*z* 1000. For MS/MS measurement, the collision energy was 19.5 eV. Data analysis was performed in the LabSolutions workstation for LCMS-8045.

### Effects of Different Reaction Parameters on HMF Elimination and HMZ Formation

A one-factor-at-a-time design was applied to estimate the effects of different reaction parameters on HMF elimination by ZGR, as well as the adduct formation between HMF and ZGR. ZGR/HMF in the molar ratio of 2: 1 was mixed in a three-necked flask and incubated at 40°C for different durations (0.5, 1.0, 1.5, 2.0, 2.5, and 3.0 h) to investigate the effect of reaction time. ZGR/HMF in the molar ratio of 2: 1 was mixed and reacted for 3 h at different temperature levels (20, 30, 40, 50, 60, and 70°C) to study the effect of temperature. In addition, temperature and reaction duration were kept constant at 40°C and 3 h, respectively, to evaluate the effect of different ZGR/HMF molar ratios (4: 1, 3: 1, 2: 1, 1: 1, and 1: 2). At the end of each reaction, 30 μL of the aqueous reaction mixture was diluted with methanol to a final volume of 500 μL for HPLC analysis. HMF and HMZ were quantified by comparing their peak areas to each standard curve.

### *In vitro* Simulation Digestion of HMZ

The adduct was subjected to *in vitro* simulated digestion at three stages, namely, the mouth, gastric, and intestinal stages, to evaluate its stability (28, 29). Briefly, HMZ (2 mg) was dissolved in DMSO (200 μL), diluted with ultra-pure water to the final concentration of 0.5 mg/mL, and then mixed with 6 mL of SSF in a flask. The mixture was magnetically stirred at 100 r/min for 2 min at 37°C to mimic saliva digestion. Afterward, 10 mL of SGF was added to simulate the gastric phase, and the mixture was reacted for 2 h under the same condition. Subsequently, 20 mL of SIF was added for the simulation of intestinal digestion for another 2 h. Samples (100 μL) were withdrawn at the end of each stage of the digestion process for HPLC analysis. Each digestion process was repeated in triplicate, and the content of HMZ was calculated by referring to the calibration curve.

### Caco-2 Cell Culture

The Caco-2 cell line (ATCC HTB-37) was obtained from the American Type Culture Collection (Rockville, MD, USA). Cells were cultivated by the method of Kosińska-Cagnazzo et al. (30) with slight modifications. Specifically, α-MEM medium supplemented with 10% FBS, 1% penicillin/streptomycin, and 1% NEAA was used to cultivate Caco-2 cells at 37°C and 5% (v/v) CO_2_ atmosphere. When the cells reached 80–90% confluence, they were detached with 0.25% trypsin-EDTA and further sub-cultured on fresh growth medium. Cells between passages 30 and 35 were used.

### Cell Viability Assay

Cell viability was measured by the CCK-8 method as described by Zhao et al. (31) with modifications. Briefly, Caco-2 cells at a density of 2.5 × 10^4^ cells/well were seeded into 96-well plates and maintained at 37°C under a humidified atmosphere containing 5% CO_2_ and 95% air for 24 h. Subsequently, the origin culture medium was removed, and different concentrations (2, 4, 8, 16, 32, and 64 mM) of HMZ and HMF were added. Three duplicate wells were set for each concentration. The cells were further incubated for 24 and 48 h, respectively. Then, 10 μL of CCK-8 solution was added into each well for 2 h, and the absorbance was read at 450 nm on a microplate reader (Multiskan, Thermo, USA).

### Annexin V and Propidium Iodide (PI) Double-Staining Assay

The Annexin V-FITC apoptosis detection kit was used to measure the percentage of apoptotic cells in flow cytometry (FACS Calibur Becton Dickinson, USA) (14). Two milliliters of each cell suspension (9 × 10^5^ cells/well) were cultured in 6-well plates. When the confluence reached 90%, the cells were treated with HMF (0, 2, 4, 8, and 16 mM) and HMZ (0, 4, 8, 16, and 32 mM) for 48 h and then harvested with trypsin (without EDTA). Subsequently, each cell suspension was centrifuged and washed with cold PBS twice. Then, the cells were suspended with 400 μL of binding buffer to a final density of 1 × 10^6^ cells/well, incubated with 5 μL of Annexin V and 1 μL of PI for 5 min in the dark, and subjected to flow cytometry assay.

### Effect of Concentration on the Absorption of HMZ in Caco-2 Cells

HMZ absorption was evaluated according to de Oliveira et al. with slight modifications (32). Briefly, Caco-2 cells were inoculated in 6-well plates at the density of 2 × 10^4^ cells/well (2 mL) and cultured until 90% confluence at 37°C with 5% CO_2_. Then, the cells were washed thrice with 1 mL of HBSS. Another 1 mL of HBSS was added for further culture for 30 min. Ten microliters of HMZ solution (pH 7.4) at concentrations of 1–100 μM were added separately and cultivated for 2 h to allow the adduct solution to be absorbed by cells under certain condition (37°C, 5% CO_2_/95% air). The treated solution was transferred into Eppendorf tubes for HPLC analysis. The control of each sample under different concentrations was the respective sample without incubation, and each of the absorption rate of the adduct by Caco-2 cells was calculated as follows:


(1) $$\text{Absorption rate }\left(\%\right)\;=\frac{S_{1}-S_{2}}{S_{1}}\times \;100\%$$

where *S*_1_ is the peak area of the control, and *S*_2_ is the peak area of the sample incubated for 2 h.

### Statistical Analysis

All experiments were repeated thrice. Data analysis was performed using Microsoft Excel, SPSS 22.0.0.1 (SPSS, Inc., Chicago, IL), and MestReNova software (version 12.0). The results were expressed as means ± standard deviation.

## Results and Discussion

### Structural Elucidation of HMZ

The retention time (*R*_t_) of HMZ was 19.54 min with purity over 95% according to the HPLC chromatogram (Supplementary Figure 1A). The UV spectrum displayed the absorption maxima at 334 nm, which exhibited a bathochromic shift compared with those of ZGR (276 nm) and HMF (284 nm) (Supplementary Figure 1B).

**Figure 1:**
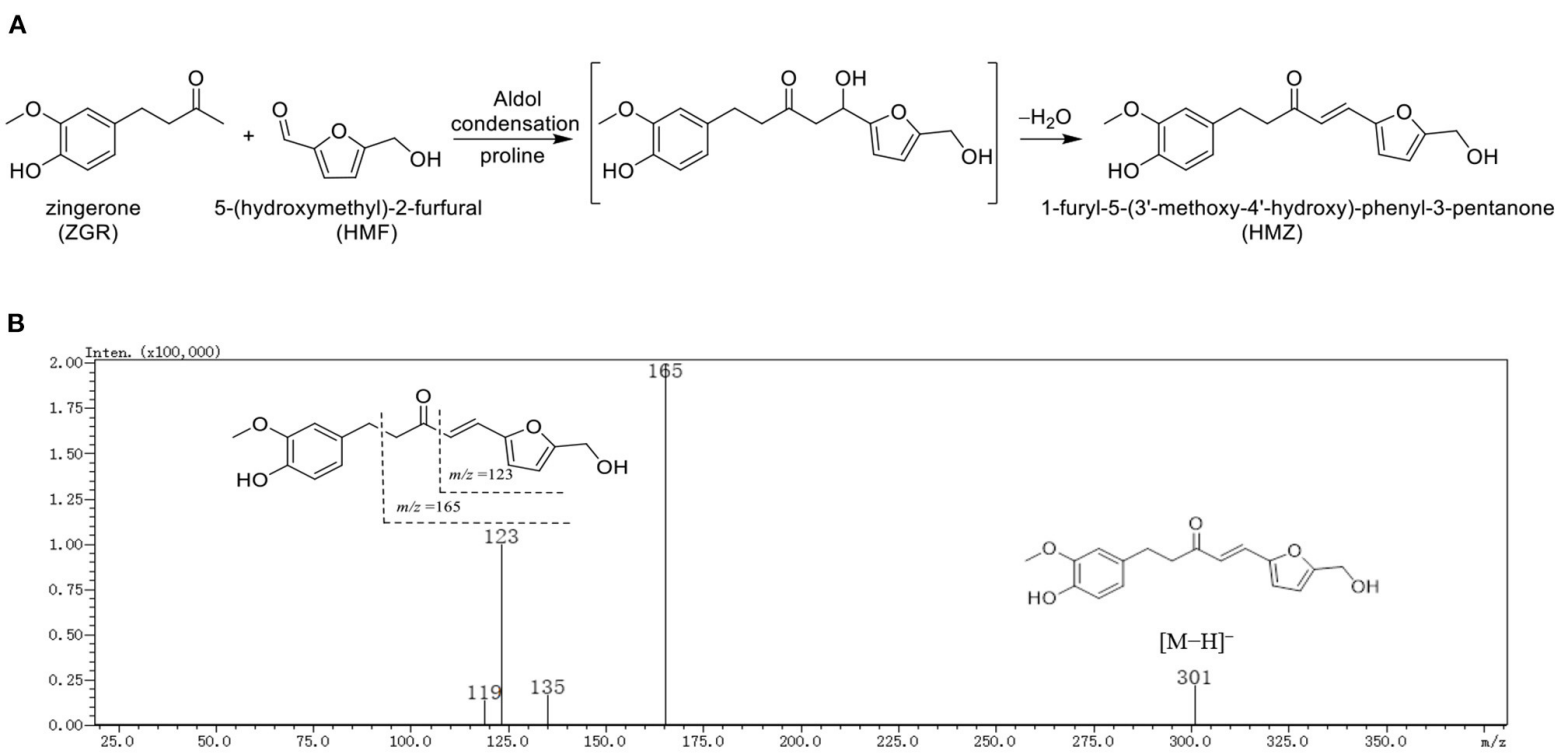
The **(A)** reaction pathway and **(B)** MS/MS spectrum of HMZ.

Based on the structural characteristic of HMF and ZGR, an aldol condensation reaction was supposed to occur between HMF and ZGR with proline as a catalyst to generate HMZ (Figure 1A). In the MS spectrum (data not shown), the adduct exhibited a deprotonated molecular ion at *m*/*z* 301 [M – H]^−^ (calculated for C_17_H_17_O_5_, 301.1), which was consistent with that of the suggested structure (C_17_H_18_O_5_). This molecular ion yielded two prominent peaks at *m*/*z* 165 and 123 in the MS/MS spectrum (Figure 1B). Specifically, the fragment ion at *m*/*z* 165 [M – C_8_H_9_O_2_]^−^ indicated the mass loss of 3-methoxy-4-hydroxybenzyl moiety, then subsequent α-scission of ketone yielded the ion at *m*/*z* 123 [M – C_8_H_9_O_2_ – C_2_H_2_O]^−^. Thus, the structure of HMZ was tentatively identified.

Subsequently, the structure of HMZ was entirely determined by the 1D and 2D NMR data, and the detailed assignment of H and C signals was listed in Table 1. The ^1^H NMR spectrum (Supplementary Figure 2) showed the signals of 7 olefinic protons (δ_H_ 7.24, 6.63 [each 1H, d, *J* = 15.8 Hz]; δ_H_ 6.81, 6.68 [each 1H, d, *J* = 8.0 Hz]; 6.71 (1H, s); 6.58, 6.36 [each 1H, d, *J* = 3.0 Hz]), one methoxy group (δ_H_ 3.85, 3H, s), and one oxygenated methylene (δ_H_ 4.63, 2H, s). The ^13^C NMR spectrum (Supplementary Figure 3) displayed 17 carbon signals, including one carbonyl (δ_C_ 199.4) and 12 unsaturated carbons (δ_C_ 157.0, 151.0, 146.6, 144.0, 133.2, 128.8, 123.2, 120.9, 116.9, 114.5, 111.3, 110.6). A detailed analysis of the 1D NMR data (Table 1) showed high similarities to those of HMF and ZGR. These results suggested that HMZ was an adduct between HMF and ZGR.

**Table 1:** ^1^H and ^13^C NMR data of HMZ, ZGR, and HMF (δ in ppm, *J* in Hz).

**Position**	**HMZ** ^a^		**ZGR** ^b^		**HMF** ^c^	
	**^1^H**	**^13^C**	**^1^H**	**^13^C**	**^1^H**	**^13^C**
1	7.24 d (15.8)	128.8			9.33 s	180.5
2	6.63 d (15.8)	123.2	2.12 s	29.5		
3	–	199.4	–	207.8		
4	2.89	30.1	2.80–2.83 t (6.6)	30.0		
5	2.89	43.5	2.70–2.74 t (6.9)	44.9		
1'	–	133.2	–	137.9		
2'	6.71 s	111.3	6.64–6.66 dd (2,8)	112.6		
3'	–	146.6	–	150.8		
4'	–	144.0	–	140.0		
5'	6.81 d (8.0)	114.5	6.80 d (8.0)	122.5		
6'	6.68 d (8.0)	120.9	6.69 d (2.0)	120.2		
7'	3.85 s	56.0	3.84 s	55.7		
2”	–	151.0			–	151.8
3”	6.58 d (3.3)	116.9			7.40 d (3.6)	126.8
4”	6.36 d (3.3)	110.6			6.55 d (3.1)	110.9
5”	–	157.0				161.3
6”	4.63 s	57.7			4.57 s	56.0

a *Measured at 400 (^1^H) and 100 (^13^C) MHz in CDCl_3_*.

b *Referred to Agarwal et al. (33),*

c *Referred to Chen et al. (34)*.

*Overlapped signals were reported without designating multiplicity*.

**Figure 2:**
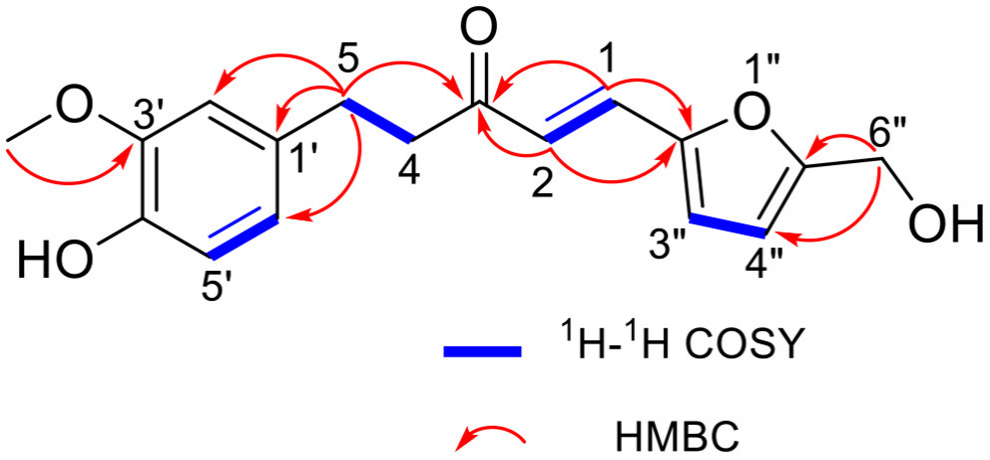
The key ^1^H-^1^H COSY and HMBC correlations of HMZ.

A further comparison of the data of HMZ with those of HMF and ZGR showed the following differences: the signal of a methyl connected with carbonyl and that of an aldehyde group were absent, two additional olefinic carbons (δ_C_ 128.8, 123.2) appeared, and some chemical shift changes (δ_C_ 199.4 in HMZ vs. δ_C_ 208.5 in ZGR; δ_C_ 157.0, 151.0, 116.9 in HMZ vs. δ_C_ 161.6, 152.3, 123.3 in HMF) occurred. These differences implied that the methyl, which was connected with the carbonyl (C-3) in ZGR, reacted with the aldehyde group of HMF to generate a double bond (C-1/C-2) to link the two residues. In the HMBC spectrum (Supplementary Figure 7), the observed correlations between H-1 (δ_H_ 7.26)/H-2 (δ_H_ 6.63) and C-3 (δ_C_ 199.4) confirmed that the double bond was directly connected with the carbonyl (C-3). The large coupling constant of H-1 and H-2 (*J* = 15.8 Hz) indicated the *E* geometry of C-1 and C-2. Thus, the exact structure of the adduct was determined as shown in Figure 2. The adduct was determined as a new compound with the systematic name of 1-furyl-5-(3′-methoxy-4′-hydroxy)-phenyl-3-pentanone.

### Effects of Different Reaction Parameters on HMF Elimination and HMZ Formation

Here, the reduction of HMF by ZGR and the formation of their adduct were investigated at different conditions, including reaction time, temperature, and molar ratio. Standard curves for the quantitative analyses of HMF (50–450 μg/mL, r^2^ = 0.9999, *n* = 3) and HMZ (50–500 μg/mL, r^2^ = 0.9999, *n* = 3) were established.

**Figure 3:**
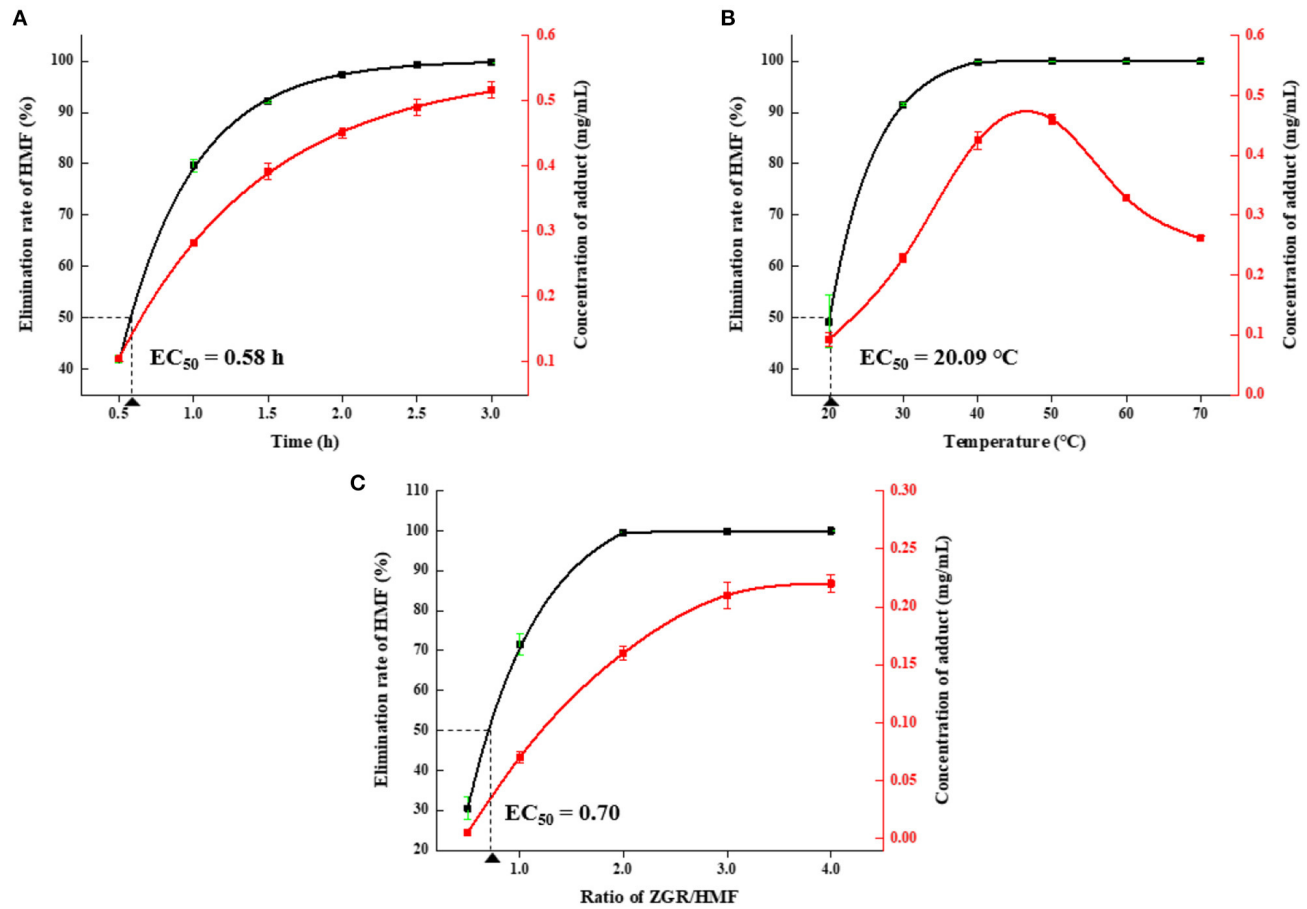
The effects of different **(A)** reaction times, **(B)** temperatures, and **(C)** molar ratios on elimination of HMF and formation of HMZ. Error bars represented the standard deviation of the average of three replicates.

As depicted in Figure 3A, the amount of HMF decreased and the concentration of HMZ increased with the prolongation of reaction time. Over 99% of HMF was depleted, and HMZ concentration reached 0.52 mg/mL after incubation for 3 h. Figure 3B showed the effect of reaction temperature on HMF reduction and HMZ formation. The remaining proportion of HMF dramatically decreased with the increase in temperature. In comparison, the amount of HMZ initially increased as the temperature increased but then decreased when the temperature was over 50°C. It was concluded that HMF elimination and HMZ formation were remarkably affected by temperature, and HMZ appeared to degrade when the temperature was over 50°C (Supplementary Figure 8). Thermal treatment-induced degradation could be also found in other substances, such as pyrethroid, which underwent hydrolysis, reduction or oxidation to yield lower molecular weight compounds (35). Figure 3C showed the influence of the molar ratio on the reduction of HMF. HMF could be remarkably trapped by ZGR when the ZGR/HMF ratio increased (1:2 to 2:1), and the elimination rate could reach as high as 99% when the ratio was ≥2:1. Additionally, a higher molar ratio promoted the accumulation of HMZ. Hence, based on the above results, the optimized condition for HMF elimination was the reaction time of 3 h, the temperature of 40°C, and the ZGR/HMF ratio of 2:1.

### Stability of HMZ in Simulated *in vitro* Digestion

Adduct formation might be accompanied by adduct ingestion, while the stability of HMZ during the digestion process was not clear. Thus, the amount of HMZ was measured after mimic digestion in the oral, gastric, and intestinal phases to evaluate its stability in *in vitro* digestion process. The content of HMZ decreased by 7.14, 14.19, and 24.06% after digestion in SSF, SGF, and SIF (Supplementary Figure 9), respectively. In addition, three new peaks with small peak areas (*R*_t_ = 12.49, 12.82, and 13.56 min) in the HPLC chromatograph could be observed in the intestinal stage as digestion progressed (Supplementary Figure 10). But regrettably, these new peaks could not be characterized with current data. While, few peaks could be observed in the other two digestive environments. The reason might be that the content of decomposition products in the oral and gastric phases was below the detection limit of HPLC. The results indicated that the stability of HMZ was of moderate level because 75.94% of the adduct was remained after undergoing three different digestion conditions.

### Effects of HMF and HMZ on the Viability and Apoptosis of Caco-2 Cells

In order to evaluate the cytotoxicity of HMF and HMZ against Caco-2 cells, a combination of the CCK-8 method and Annexin V/PI staining assay was applied and the results were shown below.

**Figure 4:**
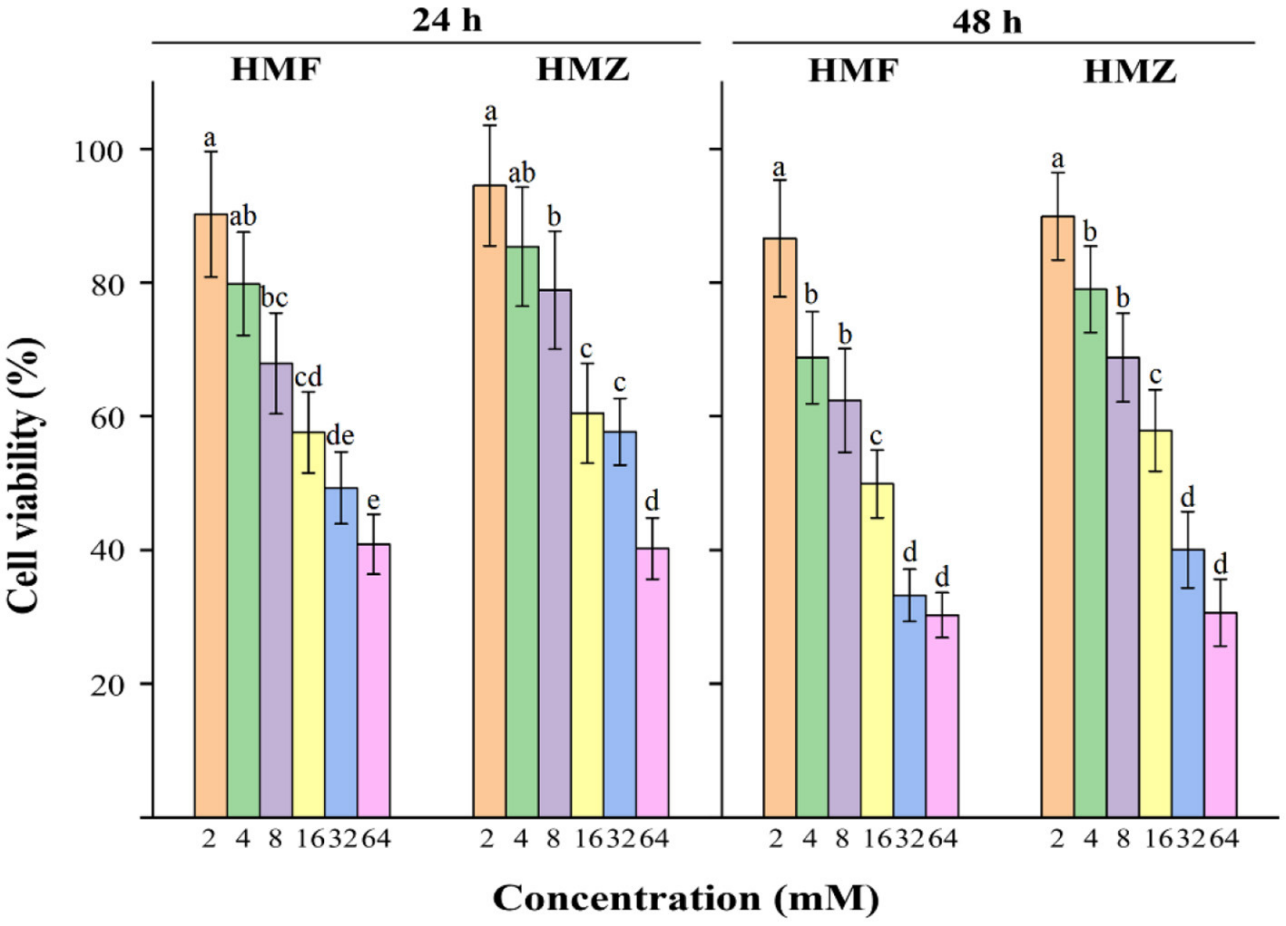
Effects of HMF and HMZ at different concentrations on cell viability after 24 and 48 h. Different letters indicated significant differences (*P* < 0.05).

In general, cells incubated with HMF and HMZ for 24 and 48 h displayed decreased viability in concentration- and time-dependent manners (Figure 4). The viability of Caco-2 cells decreased from 90.24 to 40.89% after exposure to 2–64 mM HMF for 24 h, while that of Caco-2 cells decreased from 94.54 to 40.22% in the HMZ treated group under the same condition. Similar results could be observed when the incubation time was prolonged to 48 h: the percentages of viable cells were from 86.65 to 30.27% in the HMF group and from 89.95 to 30.64% in the HMZ group. The IC_50_ values of HMZ were 41.47 ± 5.33 and 25.39 ± 3.12 mM after incubation for 24 and 48 h, respectively, whereas those of HMF were 35.39 ± 4.03 and 19.17 ± 2.10 mM. This CCK-8 analysis demonstrated that HMZ did not display obvious lower cytotoxicity to Caco-2 cells than that of HMF.

**Figure 5:**
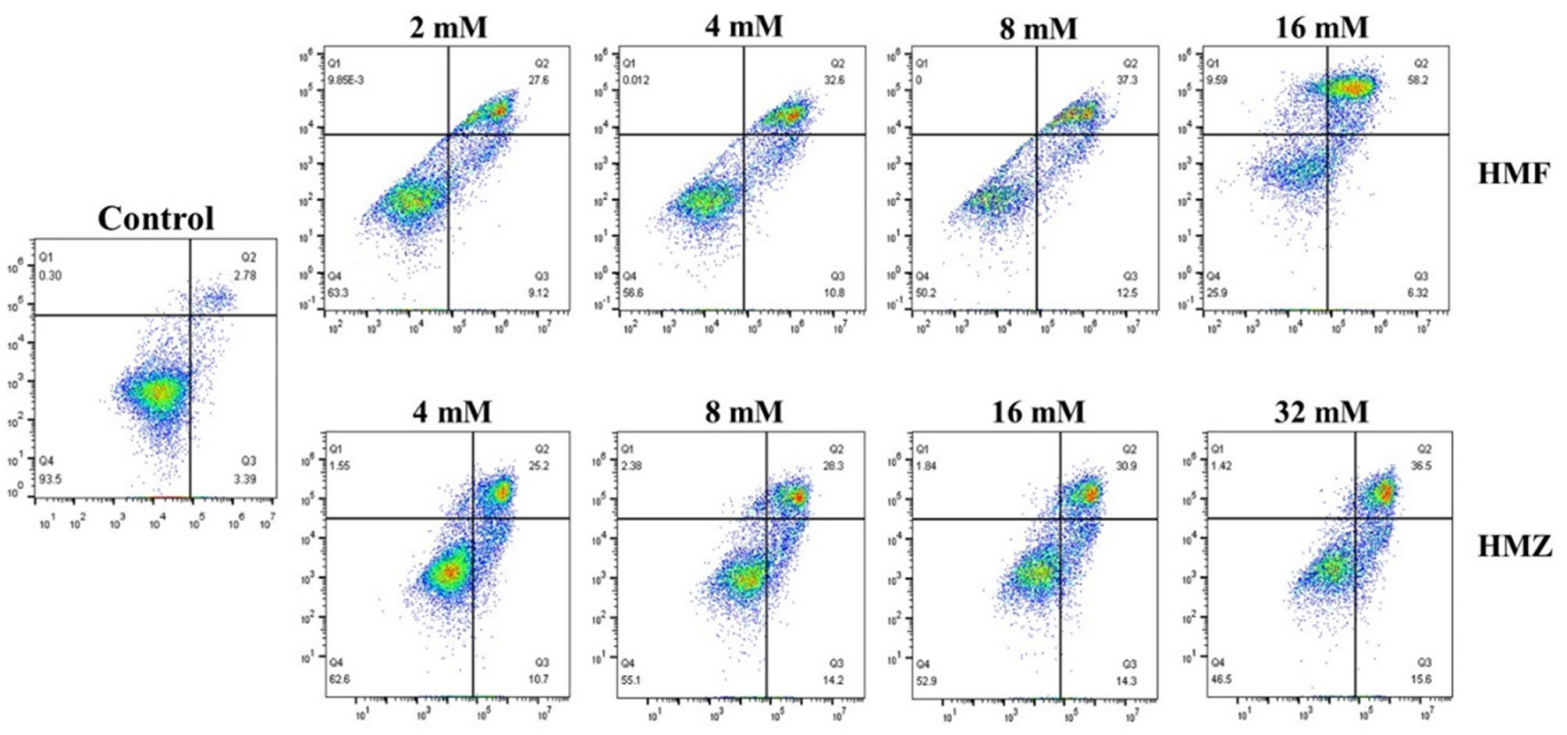
Effects of HMF and HMZ at different concentrations on apoptosis in Caco-2 cells after 48 h.

Apoptosis was commonly accompanied by some characteristic biochemical changes, such as the exposure of phosphatidylserine (Annexin V positive) on the cell membrane and the increase in cell membrane permeability that allowed the nucleic acid to be stained by PI (36). Therefore, Annexin V/PI double-staining assay was applied to measure apoptosis, and the apoptotic effects of HMF and HMZ on Caco-2 cells were determined by flow cytometry. As seen in Figure 5, HMF and HMZ induced apoptosis in Caco-2 cells dose-dependently, which manifested as an increase in the number of apoptotic cells (Annexin V+/PI– and Annexin V+/PI+). It was noticed that exposure to HMF at 2–16 mM for 48 h induced 64.52% of normal Caco-2 cells apoptosis, whereas exposure to HMZ at 4–32 mM resulted in ~52.10% of cell apoptosis. Meanwhile, compared with the control (8.54%), both HMF (43.40–64.52%) and HMZ (35.90–45.20%) displayed obvious cytotoxicity to Caco-2 cells after exposure at 4–16 mM for 48 h. Consistent with the analysis of the CCK-8 assay, the reduction of viable cells induced by HMZ was observed. In this sense, the cytotoxicity of HMZ toward Caco-2 cells should be noted, and that of HMZ to other cells was also worth studying.

### Effect of Concentration on HMZ Absorption in Caco-2 Cells

According to the HPLC analysis (Supplementary Table 1), a positive correlation was observed between the concentration of HMZ and its absorption by Caco-2 cells. While, the HMZ absorption rate by Caco-2 cells initially increased when the HMZ concentration increased from 1 to 4 μM, but declined at higher HMZ concentrations. The percentages of absorption rate were 60.74, 71.38, 56.11, and 34.71% for 1, 4, 20, and 100 μM, respectively. Although the uptake of HMZ by Caco-2 cells increased with the increase in concentration, it was observed that the absorptivity decreased at higher molar concentrations. It had been well-elucidated that compounds either lipophilic or hydrophilic could be absorbed via active and passive transport routes (37). Moreover, several intestinal efflux and uptake transporters expressed in Caco-2 cells were found to be responsible for regulating the permeation of the substrate during absorption, which would be also some factors to affect the absorption process (38).

## Conclusion

In summary, HMF could be eliminated by ZGR through the formation of an adduct via aldol condensation with proline as a catalyst. This process was not reported previously. In this work, the structural elucidation of the adduct by HPLC, MS, and NMR data, along with the investigation of the effects of different reaction conditions, namely, reaction time, temperature, and molar ratio, on HMF elimination and HMZ formation, were carried out. Besides, the stability of the adduct (HMZ) after *in vitro* digestion, as well as its absorption and cytotoxicity in Caco-2 cells, were evaluated. The results demonstrated that HMZ was stable at a moderate level. Although compared with HMF, HMZ did show a certain toxic effect against Caco-2 cells, its effect on other cells remained to be studied. Overall, this study provided new data on ketones in food materials that could contribute to the elimination of HMF.

## Data Availability Statement

The original contributions presented in the study are included in the article/Supplementary Materials, further inquiries can be directed to the corresponding author.

## Author Contributions

YK: conceptualization, methodology, and writing—original draft. ZY: methodology, investigation, and writing—original draft. NC: methodology. PC: data curation, writing, and editing. JL: supervision. SO: conceptualization. GL: conceptualization and funding acquisition. All authors contributed to the article and approved the submitted version.

## Funding

This work was supported by grants from Natural Science Foundation of China (82003609), Natural Science Foundations of Guangdong Province (2021A1515010110 and 2020A1515110453), China Postdoctoral Science Foundation (2019M663403), Graduate Education Innovation Program Project of Guangdong Province (2020JGXM107), Foundation of Guangdong Second Provincial General Hospital (TJGC-2021011), Doctoral Workstation Foundation of Guangdong Second Provincial General Hospital (2021BSGZ017), and Guangdong Basic and Applied Basic Research Foundation (2021A1515110430).

## Conflict of Interest

The authors declare that the research was conducted in the absence of any commercial or financial relationships that could be construed as a potential conflict of interest.

## Publisher's Note

All claims expressed in this article are solely those of the authors and do not necessarily represent those of their affiliated organizations, or those of the publisher, the editors and the reviewers. Any product that may be evaluated in this article, or claim that may be made by its manufacturer, is not guaranteed or endorsed by the publisher.

## Abbreviations

HMF: 5-(hydroxymethyl)-2-furfural
ZGR: zingerone
SMF: 5-sulfo-oxymethylfurfural
α-MEM: minimum essential medium ALPHA
FBS: fetal bovine serum
NEAA: non-essential amino acids
CCK-8: cell counting kit-8
PI: propidium iodide
NMR: nuclear magnetic resonance.
