# Supplementary material for: Formation and Identification of a 5-(Hydroxymethyl)-2-Furfural-Zingerone Condensate and Its Cytotoxicity in Caco-2 Cells
Source: Front Nutr. 2022 Apr 28;9:893991. doi: 10.3389/fnut.2022.893991 (PMC9106797; doi:10.3389/fnut.2022.893991)
Supplement: Supplementary file 1 [file Data_Sheet_1.docx]

Supplementary Material

## List of Supplementary Material

**Supplementary Figure 1.** HPLC chromatographs and UV spectra of HMZ, ZGR and HMF.

**Supplementary Figure 2.** ^1^H NMR spectrum of HMZ in CDCl_3_.

**Supplementary Figure 3.** ^13^C NMR spectrum of HMZ in CDCl_3_.

**Supplementary Figure 4.** DEPT 135 spectrum of HMZ in CDCl_3_.

**Supplementary Figure 5.** ^1^H-^1^H COSY spectrum of HMZ in CDCl_3_.

**Supplementary Figure 6.** HSQC spectrum of HMZ in CDCl_3_.

**Supplementary Figure 7.** HMBC spectrum of HMZ in CDCl_3_.

**Supplementary Figure 8.** The HPLC chromatographs of single factor experiment with temperature at 50℃ (A) and 60℃ (B).

**Supplementary Figure 9.** The percentage of remaining HMZ in the three stages of *in vitro* digestion.

**Supplementary Figure 10.** The HPLC chromatographs of HMZ after simulated digestion in mouth (A), gastric (B) and intestinal (C) stages.

**Supplementary Table 1.** The effects of HMZ concentration on absorption in Caco-2 cells.

## Supplementary Figure 1. HPLC chromatographs and UV spectra of HMZ, ZGR and HMF.


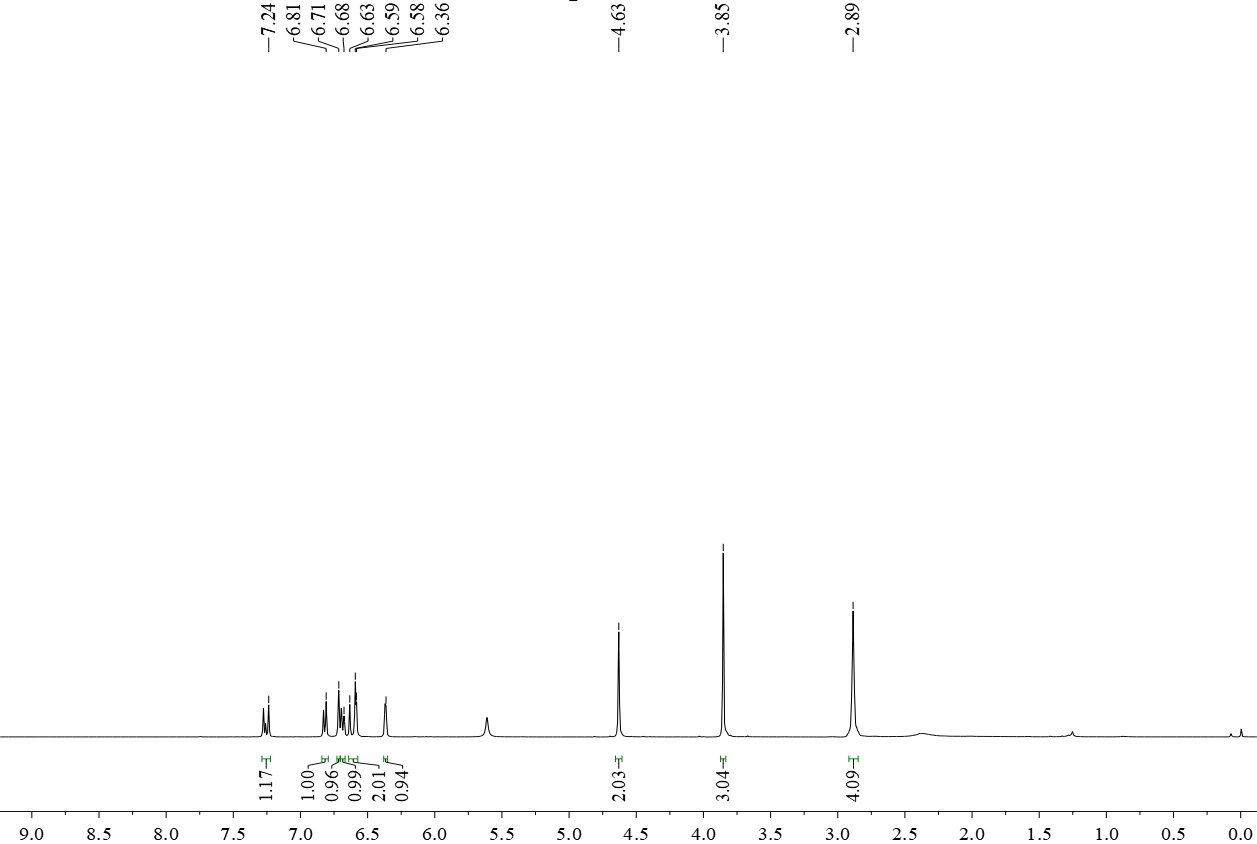


## Supplementary Figure 2. ^1^H NMR spectrum of HMZ in CDCl_3_.


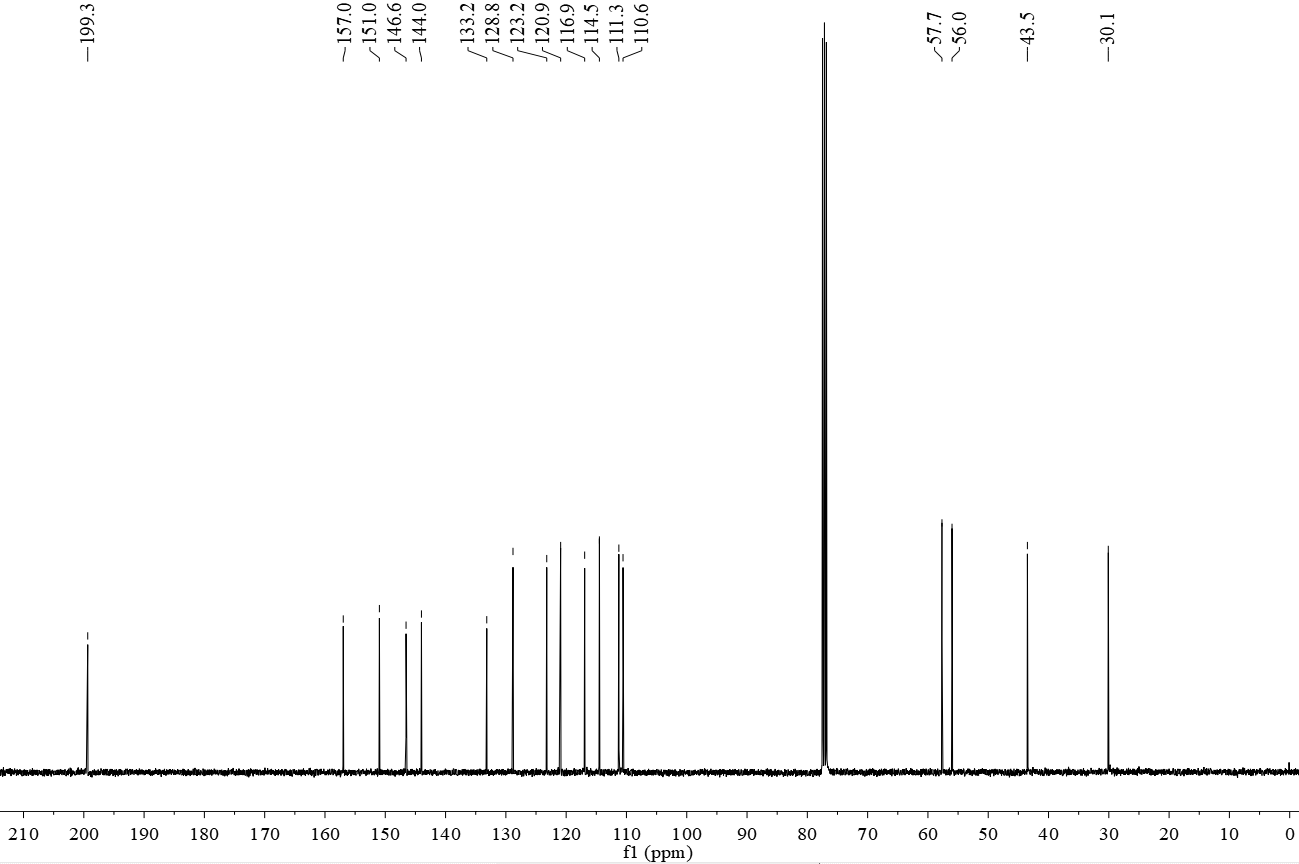


## Supplementary Figure 3. ^13^C NMR spectrum of HMZ in CDCl_3_.


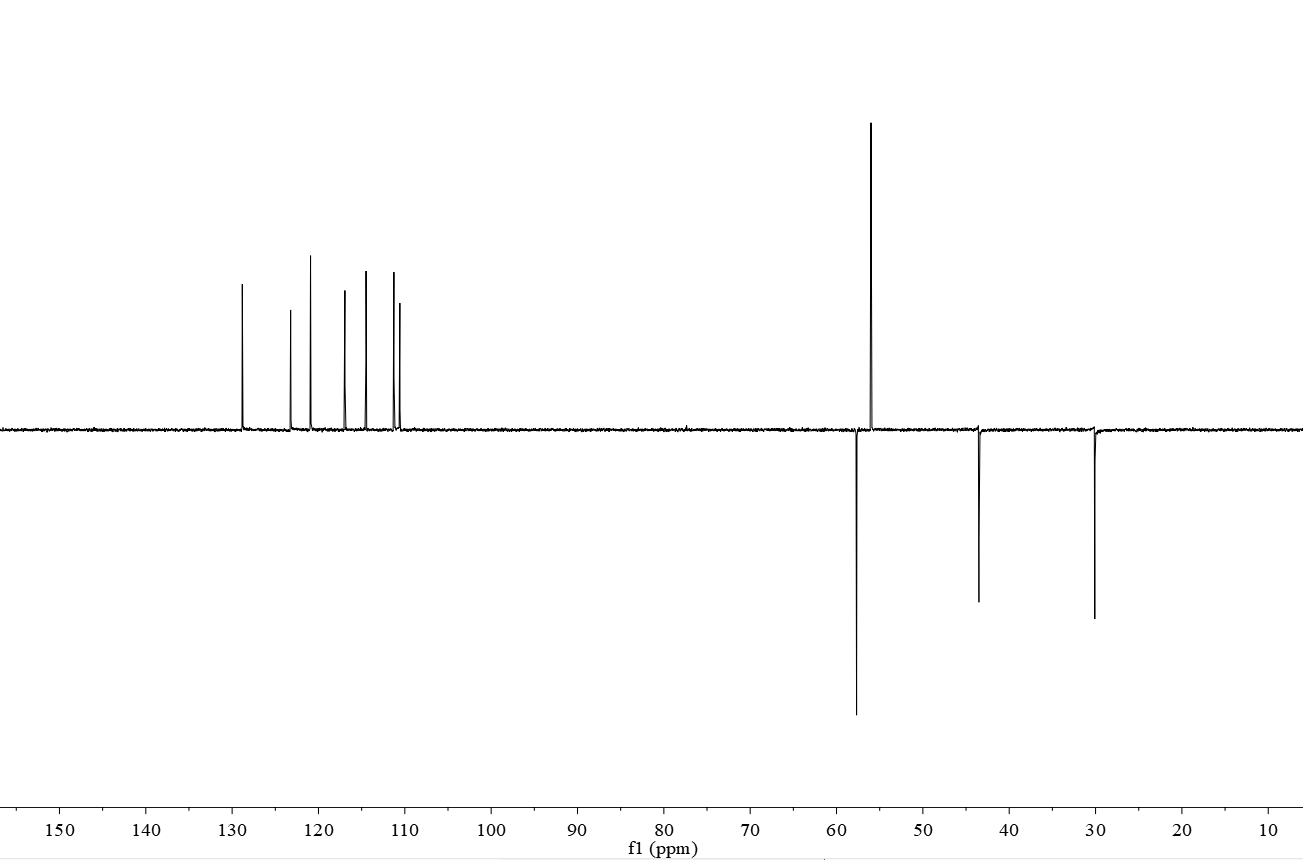


## Supplementary Figure 4. DEPT 135 spectrum of HMZ in CDCl_3_.


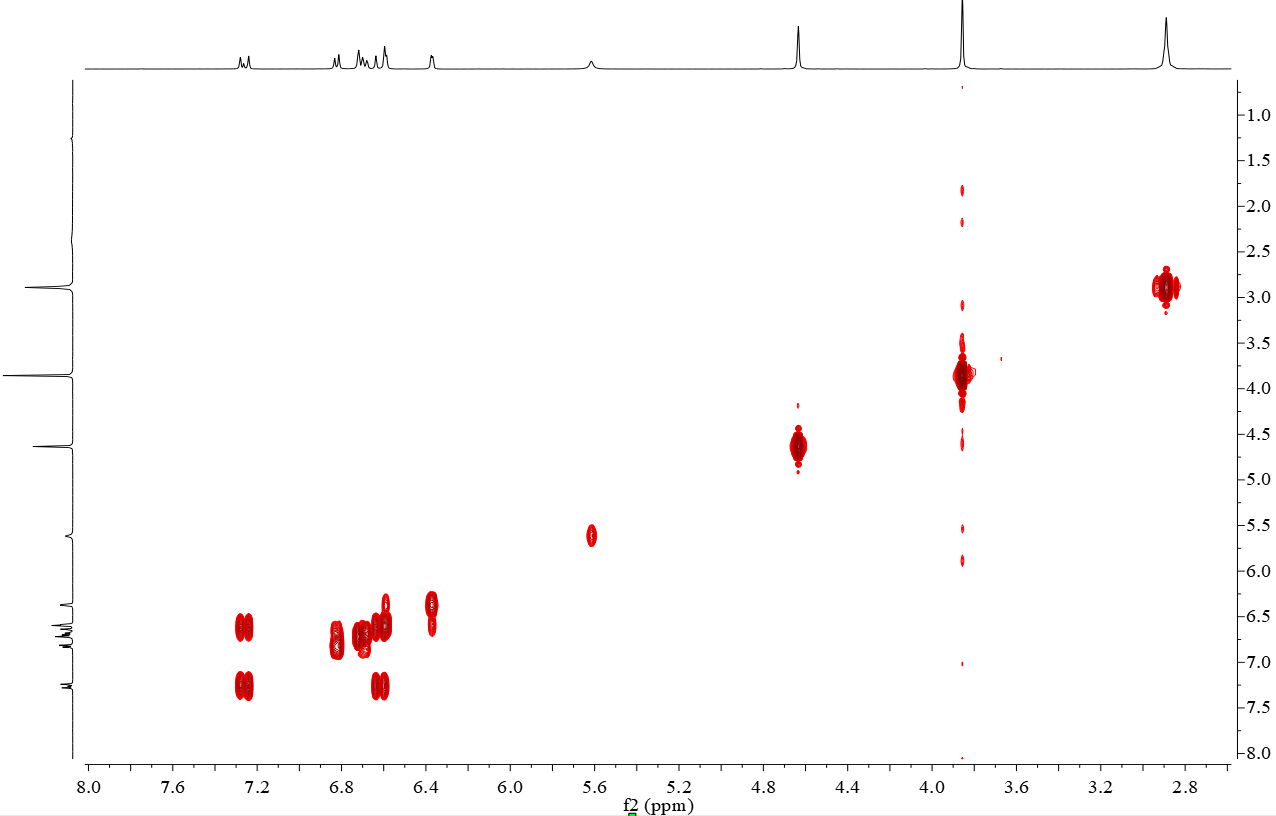


## Supplementary Figure 5. ^1^H-^1^H COSY spectrum of HMZ in CDCl_3._


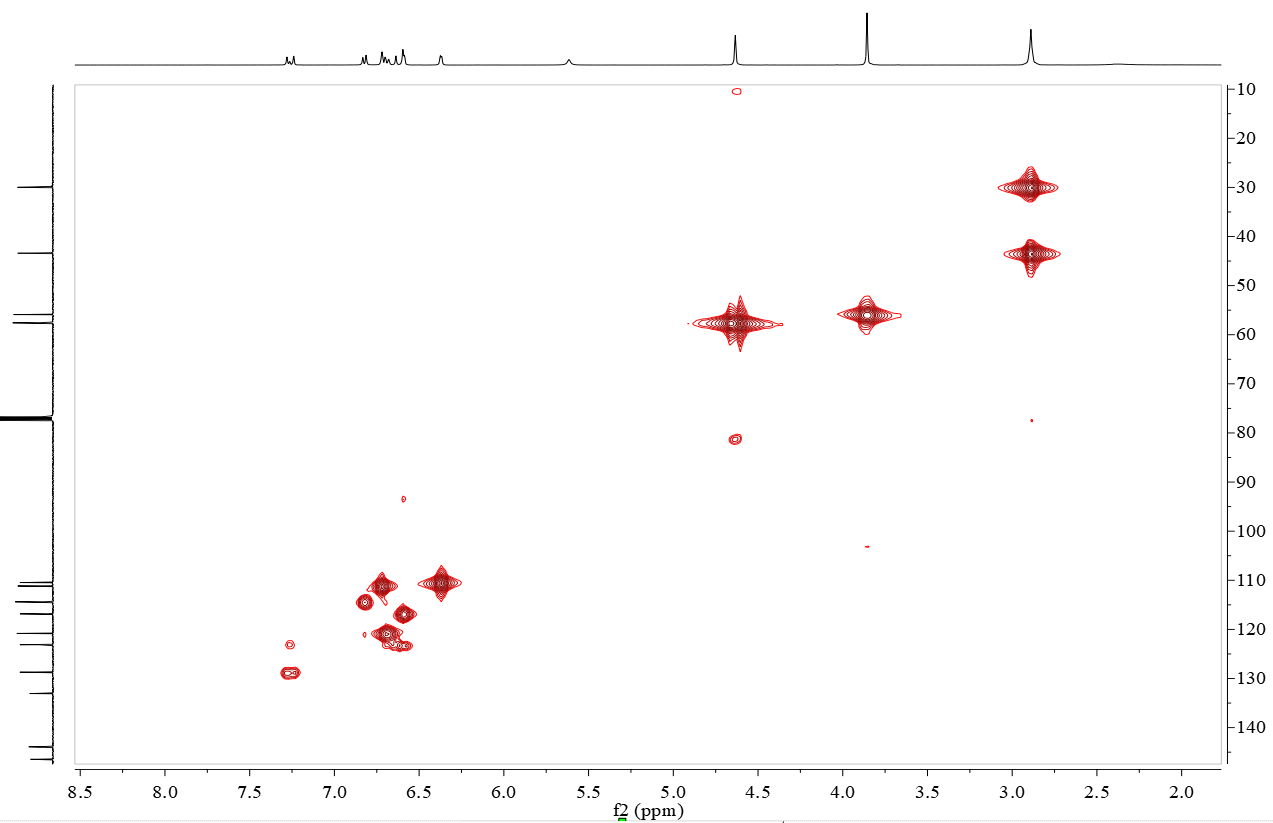


## Supplementary Figure 6. HSQC spectrum of HMZ in CDCl_3_.


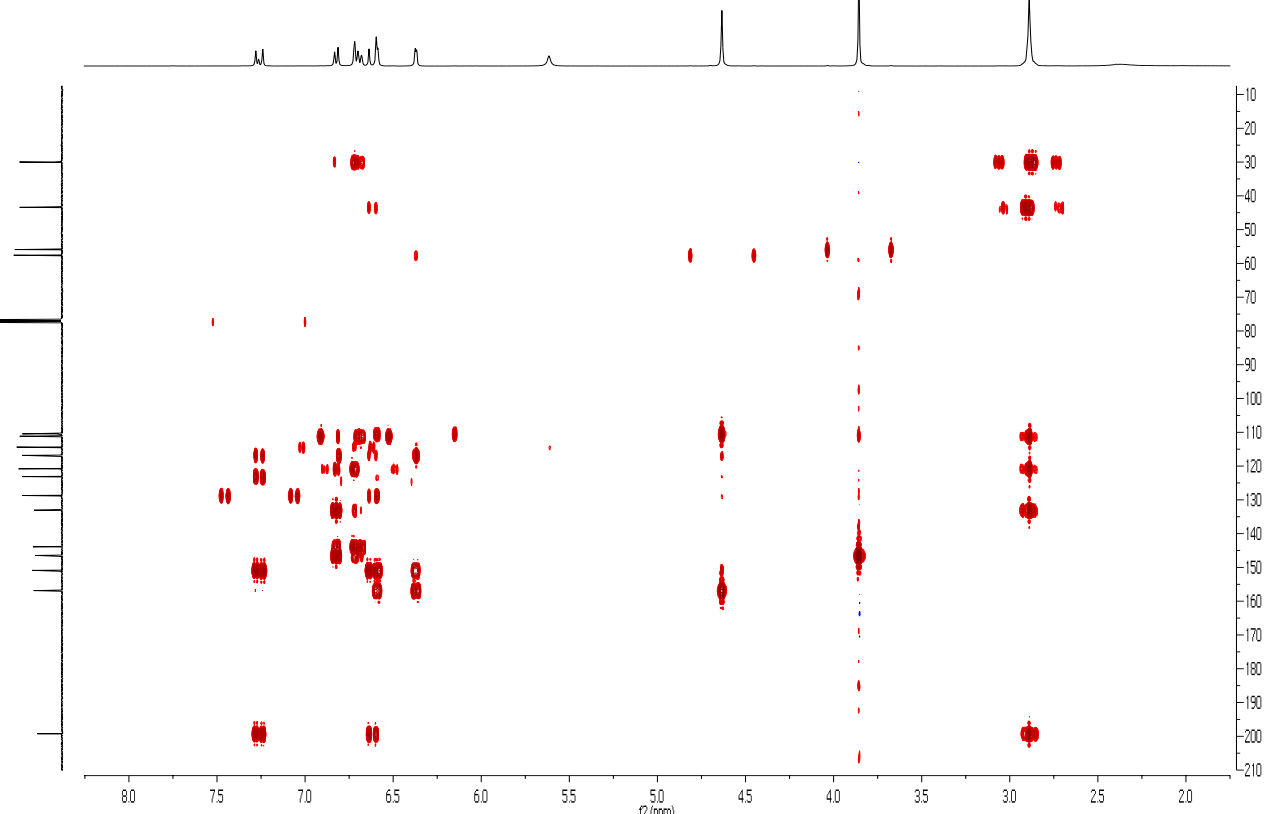


## Supplementary Figure 7. HMBC spectrum of HMZ in CDCl_3_.

## Supplementary Figure 8. The HPLC chromatographs of single factor experiment with temperature at 50℃ (A) and 60℃ (B).

**Supplementary Figure 9.** The percentage of remaining HMZ in the three stages of *in vitro* digestion.

## Supplementary Figure 10. The HPLC chromatographs of HMZ after simulated digestion in mouth (A), gastric (B) and intestinal (C) stages.

**Supplementary** **Table 1.** The effects of HMZ concentration on absorption in Caco-2 cells ^a^.

| HMZ molarity (*μ*M) | absorption (×10^-5^) (*μ*mol) | absorption rate (%) |
| --- | --- | --- |
| 1 | 0.61 ± 0.75 | 60.74 ± 7.51 |
| 4 | 2.85 ± 0.03 | 71.38 ± 0.80 |
| 20 | 11.22 ± 0.03 | 56.11 ± 1.85 |
| 100 | 34.71 ± 0.17 | 34.71 ± 1.68 |
| ^a^ Results were expressed as mean ± standard deviation (n = 3). | | |
